# Modulation of LPS-associated virulence activity for reduction of periodontal inflammatory burden

**DOI:** 10.3389/fmicb.2026.1728315

**Published:** 2026-01-30

**Authors:** Anbo Dong, Markku Lehto, Jukka Putaala, Susanna Paju, Pirkko Pussinen, Svetislav Zaric

**Affiliations:** 1 Centre for Host-Microbiome Interactions, Faculty of Dentistry, Oral & Craniofacial Sciences, King's College London, London, United Kingdom; 2 Folkhälsan Institute of Genetics, Folkhälsan Research Center, Helsinki, Finland; 3 Department of Nephrology, University of Helsinki and Helsinki University Hospital, Helsinki, Finland; 4 Research Program for Clinical and Molecular Metabolism, Faculty of Medicine Research Programs, University of Helsinki, Helsinki, Finland; 5 Department of Neurology, Helsinki University Hospital and University of Helsinki, Helsinki, Finland; 6 Department of Oral and Maxillofacial Diseases, University of Helsinki, Helsinki, Finland; 7 School of Medicine, Institute of Dentistry, University of Eastern Finland, Kuopio, Finland

**Keywords:** anti-microbial peptides, cytokines, IRF, lipopolysaccharide, NF-κB, periodontitis, polymyxin B, virulence-factor modulation

## Abstract

**Background:**

Dysbiotic oral biofilms produce virulence factors, such as lipopolysaccharide (LPS), triggering and sustaining chronic inflammation in periodontal tissues. Modulation of the bioactivity of these products offers a potential novel, adjunctive approach beyond conventional periodontal therapy.

**Methods:**

Pooled saliva and subgingival biofilm samples from 324 healthy, gingivitis, and periodontitis participants were assessed for endotoxin activity using the recombinant Factor C (rFC) assay. Functional immune-stimulation was evaluated in THP-1 and THP-1 Dual cell models through NF-κB and IRF pathways activation assessment and cytokine profiling. The modulatory effects of antimicrobial peptide LL-37 and the LPS-binding compound Polymyxin B on saliva and subgingival biofilm inflammatory potential were assessed in the same models.

**Results:**

Recombinant Factor C assays demonstrated marked reductions in endotoxin activity of saliva and subgingival biofilms treated with LL-37 and Polymyxin B (>90% reduction). Both NF-κB and IRF signaling were broadly attenuated following modulation, with polymyxin B exerting greater suppression of NF-κB activity, while LL-37 showed stronger inhibition of IRF, particularly in salivary samples. Pro-inflammatory cytokines secretion by THP-1 cells (IL-1β, IL-6, IL-8, and TNF-α) challenged with bio-modulated samples decreased by 40–75% compared to untreated samples. Interestingly, anti-inflammatory cytokines such as TGF-β and IL-10 remained largely unchanged, suggesting selective suppression of the cytokine cascade.

**Conclusion:**

Modulating LPS-associated virulence activity substantially reduces the inflammatory potential of saliva and subgingival plaque. LL-37 and Polymyxin B illustrate complementary strategies for LPS modulations and highlight the feasibility of their use as adjunctive approaches for the prevention and treatment of periodontal diseases.

## 1 Introduction

Periodontitis is one of the most prevalent chronic inflammatory conditions worldwide, contributing to tooth loss, impaired oral function, and an increased risk of systemic disorders such as diabetes and cardiovascular disease (Tonetti et al., 2017; Nascimento et al., 2024). The development of gingivitis and periodontitis reflects a dynamic interplay between oral biofilms and the host immune system (Cekici et al., 2014). Under health conditions, biofilms are part of a balanced ecosystem that supports tissue stability and regulates immune activity. When this balance is disrupted, the biofilm undergoes ecological changes that increase the release of pro-inflammatory and tissue-degrading mediators, driving persistent inflammation and progressive destruction of periodontal tissues (Kinane et al., 2017; Hajishengallis and Lamont, 2021).

Among the various mediators released by dysbiotic oral biofilms, lipopolysaccharide (LPS) plays a key role in linking local microbial dysbiosis to destructive host immune responses and systemic diseases (Pussinen et al., 2022). LPS, a structural component of the Gram-negative bacterial cell wall, has diverse immunostimulatory properties that are strongly influenced by structural variations within the lipid A domain (Jerala, 2007; Steimle et al., 2016). These differences affect the activation of downstream signaling pathways, including nuclear factor-κB (NF-κB) and interferon regulatory factors (IRFs), which drive the production of pro-inflammatory cytokines, chemokines, and tissue-degrading enzymes (Kusiak and Brady, 2022).

Monocytes are among the earliest innate immune cells to respond to biofilm challenges. As part of the host defense, they detect LPS and other biofilm-derived signals through multiple recognition pathways and initiate a rapid inflammatory response. Upon activation, monocytes release key mediators such as interleukin-1β (IL-1β), tumor necrosis factor-α (TNF-α), and interleukin-6 (IL-6), which help to coordinate pathogen clearance and recruit additional immune cells (Kusiak and Brady, 2022; Parihar et al., 2010). While these responses are critical for early defense, sustained or excessive activation disrupts tissue homeostasis and drives chronic inflammation, contributing to the progressive destruction of periodontal tissues (Yin et al., 2022). Current therapeutic strategies for periodontitis focus on mechanical biofilm disruption or antimicrobial approaches to reduce bacterial load (Yang et al., 2021). While these approaches are effective at lowering overall biofilm biomass, they do not directly neutralize endotoxin molecules, such as LPS. Critically, LPS activity is determined not only by its abundance but also by its chemical composition, particularly lipid A structure, and can therefore persist and sustain destructive immune signaling even after bacterial killing. This underscores the need for adjunctive strategies that target the bioactivity of pathogenic products rather than the microorganisms alone (Kumar et al., 2024). More importantly, extensive reliance on antibiotics has contributed to the growing global burden of antimicrobial resistance (Salam et al., 2023).

Although some experimental studies have explored the use of bioactive peptides, such as LL-37, or pharmacological agents, such as Polymyxin B, to bind and neutralize LPS (Win Maung et al., 2023; Goode et al., 2021; Hu et al., 2014; Scott et al., 2011), these approaches have not been systematically evaluated using complex clinical, biofilm-derived samples. As a result, the efficacy of direct LPS neutralization in a clinically relevant biofilm environment remains largely unknown. THP-1 monocytes and THP-1 Dual reporter cells were therefore used as established innate-immune models capable of quantifying cytokine production together with NF-κB and IRF pathway activation in response to biofilm-derived LPS (Parihar et al., 2010; Widdrington et al., 2018).

This study investigated two bio-modulatory agents, LL-37 and Polymyxin B, which neutralize LPS. A dual-assessment framework was employed to evaluate their effects. Recombinant Factor C (rFC)-based assays were used to quantify endotoxin activity of saliva and subgingival biofilm samples, while both THP-1 monocytes and THP-1 Dual reporter cells were utilized to determine the functional consequences of LPS neutralization on host immune activation, including inflammatory signaling and cytokine release. The overarching objective was to evaluate the capacity of LL-37 and Polymyxin B to neutralize LPS activity, and determine whether such modulation leads to measurable reductions in host immune responses to subgingival plaque and saliva samples, thereby informing the development of virulence-factor-based modulation strategies in periodontal care.

## 2 Materials and methods

Saliva and subgingival biofilm samples were collected from 324 participants classified as healthy, gingivitis, or periodontitis (SECRETO-Oral cohort), as described in Leskelä et al. (2024) and Putaala et al. (2017). Briefly, Participants were examined using a full-mouth periodontal assessment, and diagnostic categories were assigned according to the 2017 World Workshop Classification. Periodontal health comprised clinical periodontal health or localized gingivitis with a full-mouth bleeding score (FMBS) ≤ 30% and no radiographic bone loss. Gingivitis was defined as FMBS > 30% in the absence of clinical attachment loss or radiographic bone loss. Periodontitis was diagnosed and staged based on the presence and extent of interproximal attachment loss, radiographic bone loss, and pocketing, following the staging and grading framework.

Paraffin-stimulated saliva and subgingival biofilm samples were pooled within each diagnostic group to generate representative biological composites (Health: *n* = 52; Gingivitis: *n* = 194; Periodontitis: *n* = 78; Supplementary Figure S1). All samples were stored at −80 °C until analysis. All procedures followed the SECRETO-Oral study protocol approved by the Ethics Committees of Helsinki University Hospital (ETH11808) and Turku University Central Hospital (STE04294). Written informed consent was obtained from all participants in accordance with the Declaration of Helsinki.

THP-1 monocytes and THP-1 Dual™ reporter cells (InvivoGen, US) were cultured in RPMI-1640 medium supplemented with 10% heat-inactivated fetal bovine serum and 1% penicillin–streptomycin and then equilibrated in antibiotic-free medium before stimulation. Endotoxin activity was measured using the EndoZyme^®^ II GO recombinant Factor C assay (bioMérieux, France). For stimulation experiments, optimal working concentrations were established via preliminary titration to ensure linearity and cell viability, as detailed in Supplementary Note 1 and Supplementary Table S1. Accordingly, pooled saliva was used at a 1:500 dilution and subgingival samples at a 1:200 dilution, while commercial LPS controls from *Escherichia coli* (O111:B4, InvivoGen, US) or *Porphyromonas gingivalis* (LPS-PG, InvivoGen, US; 10 ng/mL) were applied at 10 ng/mL. Similarly, LL-37 (20 μg/mL) and Polymyxin B (50 μg/mL) were administered; these concentrations were selected based on preliminary cell viability assessments (Supplementary Table S1) and previous studies demonstrating their efficacy in neutralizing LPS-induced inflammatory responses in THP-1 cells (Scott et al., 2011; Ubanako et al., 2019). All samples were pre-incubated with inhibitors for 30 min before cell stimulation. NF-κB and IRF pathway activation were quantified after 3 h (Quanti-Blue™/Quanti-Luc™), and cytokine secretion was measured after 4 h using Luminex multiplex assays and TGF-β1 ELISA. Cell viability was confirmed using the CCK-8 assay.

Data are presented as mean ± SD of these independent replicates. Statistical analyses were performed using Python version 3.13. Differences between matched untreated and inhibitor-treated conditions were analyzed using a paired *t*-test. Comparisons between diagnostic groups are presented descriptively. To account for multiple comparisons, *p*-values were adjusted using the Benjamini-Hochberg False Discovery Rate (FDR) procedure. Results with an adjusted *p*-value of < 0.05 were considered statistically significant.

## 3 Results

### 3.1 Endotoxin activity, modulatory effects, and cytotoxicity evaluation

**Figure 1:**
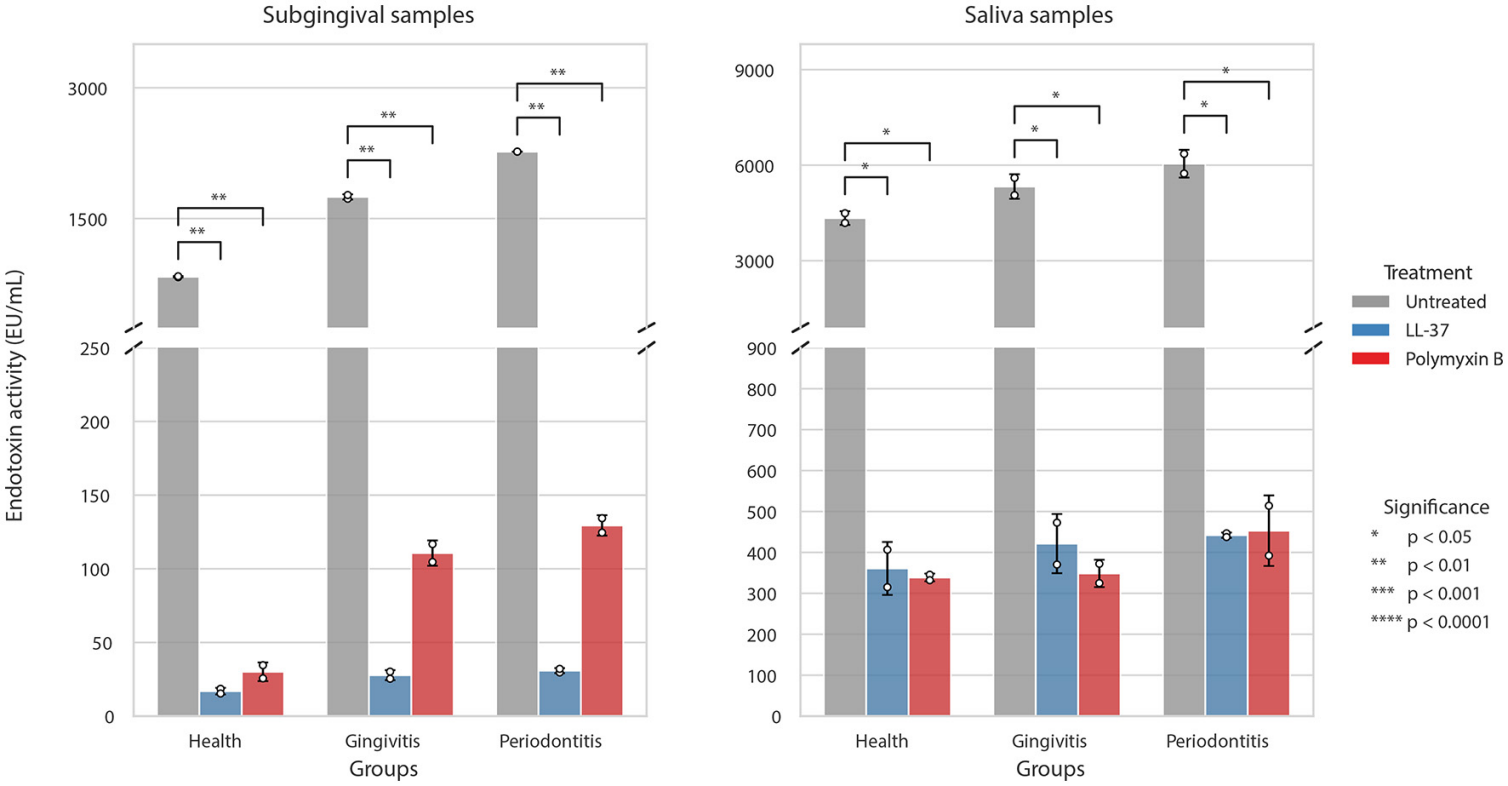
Neutralization of endotoxin activity in pooled subgingival and salivary samples by LL-37 and Polymyxin B. Data are presented as mean ± SD. Statistical significance relative to untreated controls was analyzed using a paired *t*-test followed by Benjamini-Hochberg FDR correction (*p < 0.05, **p < 0.01, ***p < 0.001, ****p < 0.0001).

Recombinant Factor C (rFC) assays quantified the endotoxin activity in each pooled sample. In subgingival plaque samples, endotoxin activity exhibited a clear progressive increase from health (833 ± 8 EU/mL) to gingivitis (1,748 ± 30 EU/mL) and periodontitis (2,268 ± 0.3 EU/mL). Similarly, in saliva, endotoxin activities ranged from 4,338 ± 215 EU/mL in health to 5,330 ± 386 EU/mL in gingivitis and 6,047 ± 437 EU/mL in periodontitis (Figure 1; Supplementary Table S2).

Following modulation, both LL-37 and Polymyxin B produced significant neutralization of endotoxin activity across all pooled samples. In subgingival biofilms, LL-37 reduced activity by >97%, while Polymyxin B achieved reductions of 93–96% (all *p* < 0.01). Similarly, in salivary samples, both inhibitors reduced endotoxin activity by 91–93% (all *p* < 0.05). These data confirm that both agents effectively neutralized endotoxin activity in all samples. Cell viability remained >90% at all tested concentrations, ensuring that the inhibitory effects were not confounded by cytotoxicity (Supplementary Table S2).

### 3.2 Activation of intracellular innate immune pathways (NF-κB and IRF)

**Figure 2:**
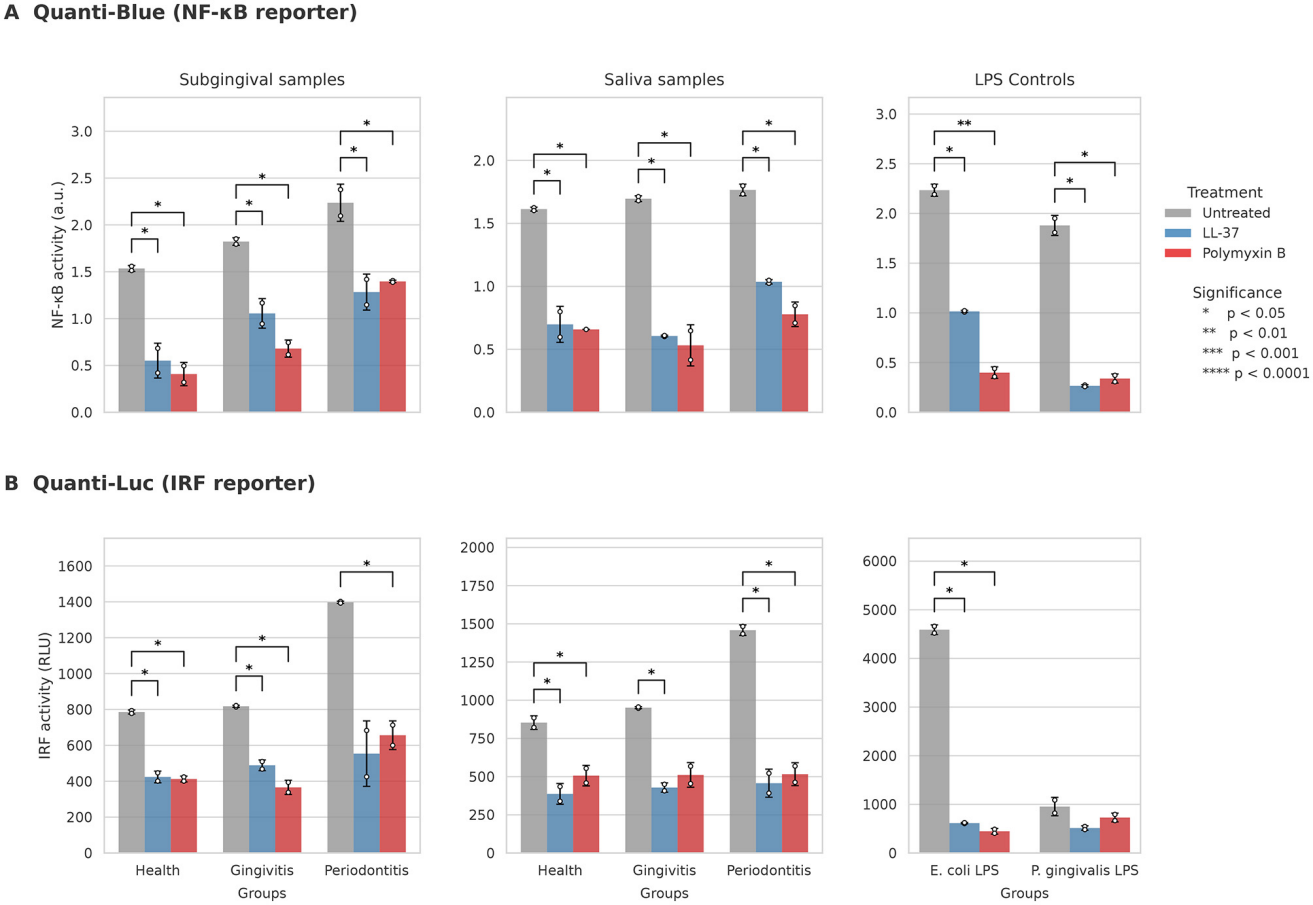
Modulation of NF-κB and IRF signaling pathways in THP-1 Dual cells by LL-37 and Polymyxin B. **(A)** NF-κB activity quantified using the Quanti-Blue assay. **(B)** IRF activity measured using the Quanti-Luc assay. Data represent mean ± SD. Statistical significance relative to the untreated condition was determined using a paired *t*-test with Benjamini-Hochberg correction for multiple comparisons (*p < 0.05, **p < 0.01, ***p < 0.001, ****p < 0.0001).

Intracellular signaling pathways, including NF-κB and IRF, were progressively activated in THP-1 Dual reporter cells upon stimulation with pooled subgingival and salivary samples, with signal intensity increasing from health to gingivitis and periodontitis (Figure 2; Supplementary Table S3). Among commercially available LPS controls, *E. coli* induced the most pronounced activation, whereas *P. gingivalis* elicited weaker responses, particularly within the IRF pathway.

In subgingival biofilms, LL-37 and Polymyxin B significantly reduced NF-κB activation by 42–64% and 37–73%, respectively (all *p* < 0.05). Polymyxin B exhibited the highest proportional inhibition in the health group. In salivary samples, LL-37 decreased NF-κB activation by 41–64% and Polymyxin B by 55–69% (all *p* < 0.05). For the purified *E. coli* LPS control, Polymyxin B suppressed NF-κB activity by 82% (*p* < 0.001).

Similarly, within the IRF pathway, LL-37 and Polymyxin B suppressed signaling in subgingival biofilms by 40–60% and 47–55%, respectively. In saliva, LL-37 reduced IRF activity by 54–68% and Polymyxin B by 40–64%. Except for subgingival periodontitis samples treated with LL-37 (*p* = 0.053) and saliva gingivitis samples modulated by Polymyxin B (*p* = 0.051), these inhibitory effects remained statistically significant in all other experimental groups (*p* < 0.05). In contrast to the clinical samples, neither inhibitor produced statistically significant reductions in *P. gingivalis* LPS-induced IRF activity (*p* > 0.05).

### 3.3 Cytokine responses following clinical samples stimulation

**Figure 3:**
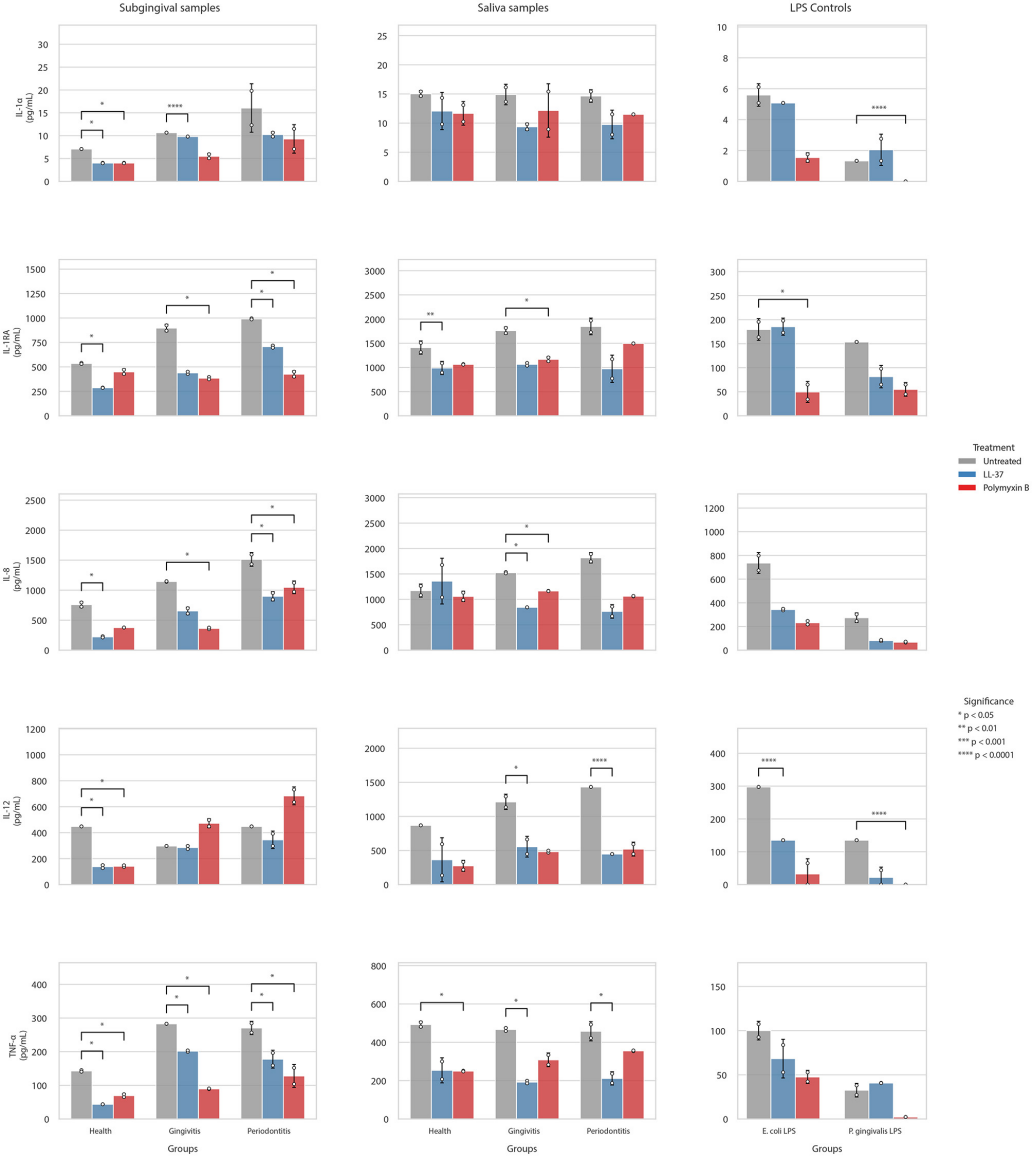
Cytokine secretion profiles in THP-1 cells stimulated with pooled clinical samples and purified LPS controls, with and without modulation by LL-37 and polymyxin B. Data are presented as mean ± SD. Statistical significance relative to the corresponding untreated condition was determined using a paired *t*-test adjusted for multiple comparisons via the Benjamini-Hochberg method (*p < 0.05, **p < 0.01, ***p < 0.001, ****p < 0.0001).

Stimulation with pooled saliva and subgingival samples induced a descriptive trend of increasing pro-inflammatory load from health to periodontitis, particularly for TNF-α, IL-1β, and IL-8 (Figure 3; Supplementary Table S4).

In subgingival samples, LL-37 and Polymyxin B provoked effective suppression of key inflammatory mediators. Both inhibitors significantly reduced TNF-α secretion by 28–69% and 51–68%, respectively. Similarly, LL-37 and Polymyxin B reduced IL-8 levels by 40–70% and 30–68%, respectively, across all diagnostic groups (all *p* < 0.05). Parallel reductions were observed for the receptor antagonist IL-1RA, which decreased by 16–57% and IL-1α (up to 50%), though these reached statistical significance only in specific conditions, such as subgingival periodontitis for IL-1RA (with Polymyxin B) and healthy samples for IL-1α (Supplementary Figure S3; Supplementary Table S4). In salivary samples, inhibition was matrix-specific: while both inhibitors significantly reduced IL-8 and TNF-α levels by 44–58% and 48–58%, respectively (all *p* < 0.05), the suppression of IL-1α and IL-1β did not reach statistical significance in most conditions (*p* > 0.05).

In contrast to the anti-inflammatory cytokines, the secretion of TGF-β remained unchanged by either inhibitor across all clinical samples (all *p* > 0.05), confirming that the observed modulation was specific to inflammatory pathways (Supplementary Figure S2; Supplementary Table S4).

Among the LPS controls, Polymyxin B achieved 100% suppression of *P. gingivalis* LPS-induced IL-12 and IL-1α secretion (all *p* < 0.001). Similarly, it significantly reduced *E. coli* LPS-induced IL-12 and IL-8 by 89% (*p* < 0.001) and 68% (*p* < 0.05), respectively.

## 4 Discussion

This study provides functional evidence that modulation of microbiome-derived virulence factors, specifically LPS, markedly attenuates the inflammatory potential of oral samples. Both LL-37 and Polymyxin B achieved profound suppression of endotoxin activity, exceeding 90% in the recombinant factor C assay, with general decreases in NF-κB and IRF activation and corresponding reductions in selective cytokine release. These findings confirm that targeted neutralization of LPS bioactivity can effectively downregulate endotoxin-driven immune activation and support the broader concept that virulence-factor modulation may alleviate dysregulated host–microbiome interactions. By linking biochemical and cellular outcomes, the present study delineates a mechanistic framework through which virulence-factor modulation achieves downstream anti-inflammatory effects *in vitro*.

Responses from untreated samples underscored the structural heterogeneity of LPS. Canonical *E. coli* lipid A strongly activated both NF-κB and IRF, whereas *P. gingivalis* LPS, enriched in atypical isoforms, produced dominant NF-κB but weak IRF activity (Jerala, 2007; Park et al., 2009). The pooled samples exhibited variable magnitudes of NF-κB and IRF activity, consistent with the dose-responsive characteristics of TLR4 signaling. As the inflammatory potential of the samples increases, the TLR4–MD2 complex engages progressively higher levels of MyD88-dependent NF-κB activation alongside the secondary TRIF-driven IRF pathway, producing the observed stepwise escalation in downstream signaling (Kagan et al., 2008; Lu et al., 2008). Similar gradients have been reported in studies of periodontal biofilm extracts and purified LPS (Al-Qutub et al., 2006; Liu et al., 2019).

Cytokine expression mirrored the activation patterns observed in the NF-κB and IRF pathways. Across pooled samples, the pro-inflammatory cytokines TNF-α, IL-1β, IL-6, IL-8, and IL-1α exhibited an increasing trend congruent with the progressive enhancement of innate immune activation (Kusiak and Brady, 2022; Van Dyke, 2017). However, TGF-β remained comparatively stable across pooled samples, which is compatible with its established role as a context-dependent regulator capable of supporting Th17-associated activity when accompanied by IL-6 (Moustakas and Heldin, 2009).

Both LL-37 and Polymyxin B substantially suppressed NF-κB and IRF activation, with widespread reductions in downstream cytokines including IL-1β, IL-6, IL-8, and TNF-α. However, their modulation profiles revealed distinct mechanistic divergences. Polymyxin B induced broader and more pronounced inhibition, particularly in contexts dominated by canonical hexa-acylated lipid A. This reflects its primary mode of action: potent physicochemical neutralization driven by high-affinity electrostatic binding and three-state accumulation at the lipid A surface (Vaara, 2019; Buchholz et al., 2024). In contrast, LL-37 exhibited greater efficacy against *P. gingivalis*-derived atypical LPS but exerted more selective, matrix-dependent modulation. Unlike Polymyxin B, LL-37 appears to engage both microbial neutralization and host-directed regulatory mechanisms, including TLR interference and intracellular signaling modulation, accounting for the sustained IRF regulation observed in saliva-stimulated systems (Scott et al., 2011; Zeth and Sancho-Vaello, 2021; Dürr et al., 2006; Mookherjee et al., 2006).

The limited modulation observed for IL-10 and TGF-β aligns with the hierarchical structure of the cytokine cascade. LL-37 and polymyxin B primarily act through extracellular LPS neutralization, attenuating NF-κB-associated cytokines such as IL-1β, IL-6, IL-8, and TNF-α. In contrast, IL-10 and TGF-β are not strictly reliant upon this acute TLR4–NF-κB axis. Their expression depends on slower SMAD-mediated transcriptional programmes, epigenetic promoter regulation and delayed autocrine feedback circuits (Moustakas and Heldin, 2009; Saraiva and O'Garra, 2010). As these pathways are not principally governed by the acute TLR4–NF-κB axis, their expression is less responsive to rapid endotoxin neutralization. The relative stability of IL-10 and TGF-β therefore reflects pathway-specific selectivity rather than incomplete inhibition, consistent with their roles as later-phase homeostatic regulators (Couper et al., 2008).

Notably, substantial endotoxin activity and inflammatory potential were observed across all diagnostic groups. This suggests that a significant underlying inflammatory burden exists in the oral cavity regardless of clinical status. These findings support a shift toward proactive virulence-factor modulation; by targeting this persistent LPS-associated virulence early, agents like LL-37 could serve as a preventive strategy to maintain oral homeostasis and forestall the transition to destructive disease.

The limitations of this study have been acknowledged. First, the use of pooled clinical samples precluded the assessment of inter-individual variability. Consequently, comparisons across diagnostic groups are descriptive in nature, as the design yields only one biological composite per group, preventing inferential statistical testing between groups. Second, due to inherent differences in sampling methods and total biomass, direct quantitative comparisons between saliva and subgingival plaque were not performed; the study quantified endotoxin activity without normalizing for bacterial load, which precludes cross-matrix standardization. Third, the study employed a focused concentration range of modulators to ensure cell viability, limiting the assessment of broader dose–response relationships. Finally, while THP-1 cells serve as a robust innate immune model, they represent a simplified *in vitro* system that lacks the complex cellular heterogeneity and structural organization of periodontal tissues found *in vivo*.

## 5 Conclusion

This study shows that modulation of oral microbiome-derived endotoxins can effectively reduce inflammatory potential of saliva and subgingival plaque. Both LL-37 and Polymyxin B markedly lowered endotoxin activity and broadly attenuated associated immune signaling and cytokine responses. These findings highlight virulence-factor neutralization as a promising adjunctive approach for controlling periodontal inflammation, warranting further validation in diverse models and clinical settings.

## Data Availability

The raw data supporting the conclusions of this article will be made available by the authors, without undue reservation.
