# Supplementary material for: Modulation of LPS-associated virulence activity for reduction of periodontal inflammatory burden
Source: Front Microbiol. 2026 Jan 30;17:1728315. doi: 10.3389/fmicb.2026.1728315 (PMC12902939; doi:10.3389/fmicb.2026.1728315)
Supplement: Supplementary file 1 [file Supplementary_file_1.docx]

Figure S1. Schematic overview of the study design and experimental workflow.

**Supplementary Note 1: Optimisation of working concentrations**

**1. Clinical Sample Input (Titration)** To determine the optimal input for clinical samples, we performed preliminary dilution-series experiments (Saliva: 1:10–1:5000; Subgingival plaque: 1:10–1:500).

- **Subgingival Samples:** A working dilution of 1:200 was selected to ensure readouts remained within the linear dynamic range while maintaining >95% THP-1 cell viability.
- **Saliva Samples:** A dilution of 1:500 was established as optimal to minimise matrix interference (viscosity and background noise) observed at lower dilutions.

**2. LPS Control Concentration** For positive controls, *E. coli* and *P. gingivalis* LPS were evaluated at concentrations consistent with previous studies (ranging from 100 pg/mL to 10 ng/mL). A final concentration of 10 ng/mL was selected as it consistently elicited a robust inflammatory response with an optimal signal-to-noise ratio in both NF-κB and IRF reporter assays.

**3. Modulator Dose Selection** Preliminary dose-optimisation indicated that lower concentrations of modulators (20 µg/mL for LL-37 and 50 µg/mL for Polymyxin B) were sufficient to achieve near-maximal neutralisation (>90% reduction) in pooled subgingival samples. Higher concentrations (50 µg/mL for LL-37 and 100 µg/mL for Polymyxin B) showed a saturation effect (plateau) without significant additional efficacy. Importantly, cell viability assays (**Table S1**) confirmed that these concentrations were non-cytotoxic, ensuring the observed plateau was due to mechanistic saturation rather than cellular compromise.

**Table S1. Impact of LL-37 and Polymyxin B on THP-1 cell viability.** Note: Cell viability was assessed using the Cell Counting Kit-8 (CCK-8) assay. THP-1 cells were incubated for 24 h with pooled subgingival plaque or saliva samples in the presence of the indicated concentrations of LL-37 (20 and 50 µg/mL) or Polymyxin B (50 and 100 µg/mL). Data are presented as mean percentage viability ± SD relative to the untreated control (normalised to 100%). No significant cytotoxicity was observed, with cell viability remaining >90% across all experimental conditions.

|  | LL37 | | Polymyxin B | |
| --- | --- | --- | --- | --- |
|  | **20 µg/ml** | **50 µg/ml** | **50 µg/ml** | **100µg/ml** |
| Subgingival | 112% (0.774) | 101% (0.554) | 107% (0.585) | 100% (0.493) |
| Saliva | **106% (0.647)** | **106% (0.579)** | **92% (0.29)** | **96% (0.359)** |

Table S2. Endotoxin activity (EU/mL) measured using the recombinant Factor C assay across subgingival and salivary pooled samples under baseline and modulated conditions. Values are presented as mean ± SD. Percentage values indicate the proportional reduction relative to the untreated condition within each pooled sample. p-values were calculated using a paired t-test comparing untreated and inhibitor-treated conditions. Adjusted p-values were derived using the Benjamini-Hochberg False Discovery Rate (FDR) procedure to account for multiple comparisons. Significance markers (*p<0.05, **p<0.01, ***p<0.001) in the adjusted columns denote statistical significance after correction.

| Sample Type | Clinical Groups | Untreated | LL-37 | |  | Polymyxin B | |
| --- | --- | --- | --- | --- | --- | --- | --- |
|  |  |  | Mean (Reduction%) | P value | Adjusted p-value | Mean (Reduction%) | Adjusted p-value |
| Saliva samples | Health | 4337.84 ± 215.31 | 360.84 ± 64.67 (91.68%) | 0.0158 * | 0.0207 * | 338.83 ± 9.44 (92.19%) | 0.0207 * |
|  | Gingivitis | 5329.90 ± 385.53 | 421.56 ± 72.38 (92.09%) | 0.0210 * | 0.0210 * | 348.64 ± 33.39 (93.46%) | 0.0207 * |
|  | Periodontitis | 6046.58 ± 436.99 | 442.11 ± 6.43 (92.69%) | 0.0173 * | 0.0207 * | 453.12 ± 86.15 (92.51%) | 0.0210 * |
| Subgingival samples | Health | 832.58 ± 7.58 | 16.95 ± 2.24 (97.96%) | 0.0027 ** | 0.0088 ** | 30.11 ± 6.32 (96.38%) | 0.0088 ** |
|  | Gingivitis | 1748.41 ± 30.17 | 27.81 ± 3.40 (98.41%) | 0.0044 ** | 0.0088 ** | 110.66 ± 8.58 (93.67%) | 0.0088 ** |
|  | Periodontitis | 2267.60 ± 0.25 | 30.88 ± 1.73 (98.64%) | < 0.001 *** | 0.0018 ** | 129.44 ± 6.92 (94.29%) | 0.0042 ** |

Table S3. NF-κB and IRF reporter activity Quanti-Blue (optical density at 405 nm) and Quanti-Luc (relative luminescence units) across subgingival and salivary samples and LPS stimulation under baseline and modulated conditions. Note: Values are presented as mean ± SD. P-values were calculated using a paired t-test comparing untreated and inhibitor-treated conditions. Adjusted p-values were derived using the Benjamini-Hochberg False Discovery Rate (FDR) procedure to account for multiple comparisons. Significance markers (*p<0.05, **p<0.01, ***p<0.001) in the adjusted columns denote statistical significance after correction.

| Assay | Sample Type | Condition | Untreated | LL37 | | | Polymyxin B | | | |
| --- | --- | --- | --- | --- | --- | --- | --- | --- | --- | --- |
|  |  |  | **Mean ± SD** | **Mean ± SD (Reduction)** | **P-value** | **Adjusted p-value** | **Mean ± SD (Reduction)** | **P-value** | **Adjusted p-value** |  |
| Quanti-Blue (SEAP) | **Subgingival samples** | **Health** | 1.54 ± 0.03 | 0.55 ± 0.19 (64.08%) | 0.0494 * | 0.0494 * | 0.41 ± 0.12 (73.43%) | 0.0185 * | 0.0296 * |  |
|  |  | **Gingivitis** | 1.82 ± 0.04 | 1.06 ± 0.16 (42.09%) | 0.0343 * | 0.0443 * | 0.68 ± 0.09 (62.69%) | 0.0102 * | 0.0203 * |  |
|  |  | **Periodontitis** | 2.24 ± 0.20 | 1.28 ± 0.19 (42.63%) | 0.0015 ** | 0.0120 * | 1.40 ± 0.01 (37.54%) | 0.0495 * | 0.0495 * |  |
|  | **Saliva samples** | **Health** | 1.61 ± 0.02 | 0.70 ± 0.14 (56.73%) | 0.0388 * | 0.0443 * | 0.66 ± 0.00 (59.21%) | 0.0037 ** | 0.0147 * |  |
|  |  | **Gingivitis** | 1.70 ± 0.02 | 0.61 ± 0.01 (64.30%) | 0.0060 ** | 0.0239 * | 0.53 ± 0.16 (68.66%) | 0.0358 * | 0.0409 * |  |
|  |  | **Periodontitis** | 1.77 ± 0.05 | 1.04 ± 0.02 (41.32%) | 0.0194 * | 0.0310 * | 0.78 ± 0.10 (55.93%) | 0.0326 * | 0.0409 * |  |
|  | **LPS Controls** | ***E. coli* LPS** | 2.23 ± 0.06 | 1.02 ± 0.01 (54.57%) | 0.0130 * | 0.0310 * | 0.40 ± 0.06 (82.14%) | < 0.001 *** | 0.0028 ** |  |
|  |  | ***P. gingivalis* LPS** | 1.88 ± 0.10 | 0.27 ± 0.01 (85.85%) | 0.0157 * | 0.0310 * | 0.34 ± 0.05 (81.94%) | 0.0081 ** | 0.0203 * |  |
| Quanti-Luc (IRF) | **Subgingival samples** | **Health** | 786.00 ± 11.31 | 424.00 ± 32.53 (46.06%) | 0.0272 * | 0.0363 * | 412.50 ± 16.26 (47.52%) | 0.0030 ** | 0.0119 * |  |
|  |  | **Gingivitis** | 818.00 ± 5.66 | 489.00 ± 29.70 (40.22%) | 0.0164 * | 0.0263 * | 366.00 ± 39.60 (55.26%) | 0.0169 * | 0.0270 * |  |
|  |  | **Periodontitis** | 1397.50 ± 6.36 | 554.00 ± 182.43 (60.36%) | 0.0466 * | 0.0533 (ns) | 656.50 ± 79.90 (53.02%) | 0.0223 * | 0.0297 * |  |
|  | **Saliva samples** | **Health** | 853.50 ± 44.55 | 387.00 ± 67.88 (54.66%) | 0.0113 * | 0.0257 * | 506.50 ± 67.18 (40.66%) | 0.0147 * | 0.0270 * |  |
|  |  | **Gingivitis** | 951.50 ± 6.36 | 428.50 ± 30.41 (54.97%) | 0.0103 * | 0.0257 * | 511.00 ± 80.61 (46.30%) | 0.0442 * | 0.0505 (ns) |  |
|  |  | **Periodontitis** | 1458.50 ± 34.65 | 457.00 ± 91.92 (68.67%) | 0.0129 * | 0.0257 * | 516.00 ± 74.95 (64.62%) | 0.0096 ** | 0.0257 * |  |
|  | **LPS Controls** | ***E. coli* LPS** | 4591.00 ± 98.99 | 615.00 ± 14.14 (86.60%) | 0.0064 ** | 0.0257 * | 447.00 ± 57.98 (90.26%) | 0.0022 ** | 0.0119 * |  |
|  |  | ***P. gingivalis* LPS** | 955.00 ± 189.50 | 514.00 ± 45.25 (46.18%) | 0.1146 (ns) | 0.1146 (ns) | 730.00 ± 90.51 (23.56%) | 0.2297 (ns) | 0.2297 (ns) |  |

**Figure S2. Supplemental cytokine profiles (IL-6, IL-10, IL-12, and TGF-β) in modulated THP-1 cells.** Data represent mean ± SD (n=2). Statistical significance relative to untreated controls was determined using a paired t-test with Benjamini-Hochberg FDR correction (*p < 0.05, **p < 0.01, ***p < 0.001, ****p < 0.0001).

Table S4. Cytokine release profiles of THP-1 cells stimulated with subgingival and salivary samples and LPS stimulation under untreated baseline and modulated conditions. Values are presented as mean ± SD (pg/mL). P-values were calculated using a paired t-test comparing untreated and inhibitor-treated conditions. Adjusted p-values were derived using the Benjamini-Hochberg False Discovery Rate (FDR) procedure to account for multiple comparisons. Significance markers (*p<0.05, **p<0.01, ***p<0.001) in the adjusted columns denote statistical significance after correction."

| Cytokine | Sample Type | Condition | Untreated | LL37 | | | Polymyxin B | | |
| --- | --- | --- | --- | --- | --- | --- | --- | --- | --- |
|  |  |  | **Mean ± SD** | **Mean ± SD (Reduction)** | **P-value** | **Adjusted p-value** | **Mean ± SD (Reduction)** | **P-value** | **Adjusted p-value** |
| IL-1alpha | **Subgingival samples** | **Health** | 7.08 ± 0.01 | 4.03 ± 0.08 (43.19%) | 0.0062 | 0.0499 * | 4.03 ± 0.08 (43.19%) | 0.0062 | 0.0499 * |
|  |  | **Gingivitis** | 10.66 ± 0.00 | 9.81 ± 0.00 (7.97%) | 0.0000 | 0.0000 **** | 5.50 ± 0.60 (48.36%) | 0.0262 | 0.0932 (ns) |
|  |  | **Periodontitis** | 16.06 ± 5.30 | 10.23 ± 0.60 (36.27%) | 0.1651 | 0.2245 (ns) | 9.29 ± 3.12 (42.12%) | 0.0715 | 0.1354 (ns) |
|  | **Saliva samples** | **Health** | 15.03 ± 0.54 | 12.07 ± 3.20 (19.69%) | 0.2318 | 0.2840 (ns) | 11.67 ± 2.02 (22.36%) | 0.0964 | 0.1543 (ns) |
|  |  | **Gingivitis** | 14.90 ± 1.76 | 9.37 ± 0.62 (37.14%) | 0.0941 | 0.1523 (ns) | 12.17 ± 4.58 (18.35%) | 0.2006 | 0.2526 (ns) |
|  |  | **Periodontitis** | 14.65 ± 1.07 | 9.77 ± 2.45 (33.34%) | 0.1503 | 0.2086 (ns) | 11.50 ± 0.00 (21.50%) | 0.0754 | 0.1354 (ns) |
|  | **LPS Controls** | **E. coli LPS** | 5.60 ± 0.73 | 5.08 ± 0.00 (9.20%) | 0.2500 | 0.2906 (ns) | 1.54 ± 0.30 (72.39%) | 0.0235 | 0.0915 (ns) |
|  |  | **P. gingivalis LPS** | 1.33 ± 0.00 | 2.04 ± 1.01 (-53.76%) | 0.7500 | 0.7907 (ns) | 0.00 ± 0.00 (100.00%) | 0.0000 | 0.0000 **** |
| IL-1RA | **Subgingival samples** | **Health** | 535.46 ± 7.47 | 287.06 ± 2.95 (46.39%) | 0.0041 | 0.0481 * | 449.55 ± 30.50 (16.04%) | 0.0596 | 0.1309 (ns) |
|  |  | **Gingivitis** | 897.62 ± 38.81 | 439.25 ± 15.95 (51.06%) | 0.0268 | 0.0932 (ns) | 385.12 ± 16.19 (57.10%) | 0.0099 | 0.0587 (ns) |
|  |  | **Periodontitis** | 991.17 ± 7.62 | 707.34 ± 15.25 (28.64%) | 0.0060 | 0.0499 * | 425.71 ± 35.09 (57.05%) | 0.0170 | 0.0722 (ns) |
|  | **Saliva samples** | **Health** | 1411.60 ± 137.14 | 988.67 ± 136.58 (29.96%) | 0.0003 | 0.0057 ** | 1063.91 ± 7.93 (24.63%) | 0.0818 | 0.1426 (ns) |
|  |  | **Gingivitis** | 1762.09 ± 75.70 | 1064.54 ± 38.81 (39.59%) | 0.0368 | 0.1021 (ns) | 1167.93 ± 52.86 (33.72%) | 0.0086 | 0.0535 (ns) |
|  |  | **Periodontitis** | 1847.80 ± 171.74 | 971.65 ± 282.10 (47.42%) | 0.0283 | 0.0932 (ns) | 1496.67 ± 0.00 (19.00%) | 0.1060 | 0.1579 (ns) |
|  | **LPS Controls** | **E. coli LPS** | 179.61 ± 22.56 | 185.41 ± 17.80 (-3.23%) | 0.8329 | 0.8581 (ns) | 49.59 ± 21.75 (72.39%) | 0.0014 | 0.0211 * |
|  |  | **P. gingivalis LPS** | 153.66 ± 0.00 | 81.48 ± 23.36 (46.97%) | 0.0716 | 0.1354 (ns) | 54.95 ± 14.16 (64.24%) | 0.0322 | 0.0932 (ns) |
| IL-8 | **Subgingival samples** | **Health** | 759.54 ± 50.10 | 222.75 ± 13.72 (70.67%) | 0.0152 | 0.0715 (ns) | 377.21 ± 1.15 (50.34%) | 0.0301 | 0.0932 (ns) |
|  |  | **Gingivitis** | 1144.28 ± 8.08 | 654.33 ± 62.27 (42.82%) | 0.0322 | 0.0932 (ns) | 362.96 ± 18.25 (68.28%) | 0.0029 | 0.0398 * |
|  |  | **Periodontitis** | 1512.83 ± 114.37 | 898.81 ± 76.55 (40.59%) | 0.0139 | 0.0684 (ns) | 1047.91 ± 105.61 (30.73%) | 0.0042 | 0.0481 * |
|  | **Saliva samples** | **Health** | 1172.95 ± 128.86 | 1358.92 ± 449.59 (-15.85%) | 0.7186 | 0.7635 (ns) | 1059.21 ± 101.37 (9.70%) | 0.3059 | 0.3438 (ns) |
|  |  | **Gingivitis** | 1524.64 ± 17.82 | 843.74 ± 0.95 (44.66%) | 0.0062 | 0.0499 * | 1165.25 ± 4.27 (23.57%) | 0.0085 | 0.0535 (ns) |
|  |  | **Periodontitis** | 1818.63 ± 102.79 | 763.04 ± 133.38 (58.04%) | 0.0499 | 0.1235 (ns) | 1062.70 ± 5.83 (41.57%) | 0.0288 | 0.0932 (ns) |
|  | **LPS Controls** | **E. coli LPS** | 736.22 ± 88.22 | 343.13 ± 9.20 (53.39%) | 0.0449 | 0.1199 (ns) | 231.83 ± 20.70 (68.51%) | 0.0482 | 0.1235 (ns) |
|  |  | **P. gingivalis LPS** | 274.23 ± 40.40 | 81.41 ± 7.01 (70.31%) | 0.0548 | 0.1307 (ns) | 68.06 ± 5.47 (75.18%) | 0.0497 | 0.1235 (ns) |
| IL-12 | **Subgingival samples** | **Health** | 448.28 ± 0.00 | 138.50 ± 15.63 (69.10%) | 0.0113 | 0.0643 (ns) | 141.75 ± 9.23 (68.38%) | 0.0068 | 0.0499 * |
|  |  | **Gingivitis** | 297.45 ± 0.00 | 285.66 ± 16.67 (3.96%) | 0.2500 | 0.2906 (ns) | 472.87 ± 34.77 (-58.97%) | 0.9557 | 0.9557 (ns) |
|  |  | **Periodontitis** | 448.28 ± 0.00 | 345.04 ± 67.31 (23.03%) | 0.1375 | 0.1948 (ns) | 683.86 ± 68.79 (-52.55%) | 0.9352 | 0.9421 (ns) |
|  | **Saliva samples** | **Health** | 868.95 ± 0.00 | 363.93 ± 323.44 (58.12%) | 0.1354 | 0.1938 (ns) | 275.43 ± 80.71 (68.30%) | 0.0305 | 0.0932 (ns) |
|  |  | **Gingivitis** | 1213.35 ± 112.14 | 555.66 ± 151.87 (54.20%) | 0.0136 | 0.0684 (ns) | 483.20 ± 20.15 (60.18%) | 0.0283 | 0.0932 (ns) |
|  |  | **Periodontitis** | 1432.50 ± 0.00 | 448.28 ± 0.00 (68.71%) | 0.0000 | 0.0000 **** | 520.46 ± 102.08 (63.67%) | 0.0251 | 0.0932 (ns) |
|  | **LPS Controls** | **E. coli LPS** | 297.45 ± 0.00 | 135.22 ± 0.00 (54.54%) | 0.0000 | 0.0000 **** | 32.61 ± 46.12 (89.04%) | 0.0390 | 0.1061 (ns) |
|  |  | **P. gingivalis LPS** | 135.22 ± 0.00 | 22.00 ± 31.11 (83.73%) | 0.0611 | 0.1309 (ns) | 0.00 ± 0.00 (100.00%) | 0.0000 | 0.0000 **** |
| TNF-alpha | **Subgingival samples** | **Health** | 142.86 ± 3.22 | 43.74 ± 0.00 (69.38%) | 0.0073 | 0.0499 * | 69.44 ± 5.82 (51.40%) | 0.0277 | 0.0932 (ns) |
|  |  | **Gingivitis** | 282.89 ± 0.25 | 201.61 ± 2.90 (28.73%) | 0.0073 | 0.0499 * | 89.62 ± 1.36 (68.32%) | 0.0013 | 0.0211 * |
|  |  | **Periodontitis** | 270.54 ± 19.70 | 177.76 ± 26.48 (34.29%) | 0.0164 | 0.0722 (ns) | 127.92 ± 34.14 (52.72%) | 0.0227 | 0.0910 (ns) |
|  | **Saliva samples** | **Health** | 492.75 ± 16.58 | 253.71 ± 65.32 (48.51%) | 0.0757 | 0.1354 (ns) | 249.86 ± 2.69 (49.29%) | 0.0129 | 0.0684 (ns) |
|  |  | **Gingivitis** | 466.80 ± 11.16 | 192.58 ± 9.39 (58.74%) | 0.0169 | 0.0722 (ns) | 308.56 ± 35.81 (33.90%) | 0.0659 | 0.1309 (ns) |
|  |  | **Periodontitis** | 457.54 ± 49.98 | 211.76 ± 34.60 (53.72%) | 0.0141 | 0.0684 (ns) | 355.39 ± 2.69 (22.32%) | 0.1113 | 0.1610 (ns) |
|  | **LPS Controls** | **E. coli LPS** | 100.06 ± 10.53 | 68.32 ± 21.86 (31.72%) | 0.1990 | 0.2526 (ns) | 47.64 ± 7.38 (52.39%) | 0.0755 | 0.1354 (ns) |
|  |  | **P. gingivalis LPS** | 32.62 ± 7.69 | 40.75 ± 0.22 (-24.92%) | 0.8167 | 0.8517 (ns) | 2.26 ± 0.00 (93.07%) | 0.0564 | 0.1309 (ns) |
| IL-1beta | **Subgingival samples** | **Health** | 40.76 ± 7.30 | 16.96 ± 1.90 (58.39%) | 0.0849 | 0.1462 (ns) | 25.59 ± 0.00 (37.21%) | 0.1045 | 0.1579 (ns) |
|  |  | **Gingivitis** | 72.61 ± 11.42 | 69.09 ± 1.26 (4.84%) | 0.3552 | 0.3959 (ns) | 36.09 ± 0.71 (50.29%) | 0.0651 | 0.1309 (ns) |
|  |  | **Periodontitis** | 81.23 ± 2.93 | 69.48 ± 0.45 (14.47%) | 0.0640 | 0.1309 (ns) | 53.94 ± 5.23 (33.61%) | 0.0189 | 0.0778 (ns) |
|  | **Saliva samples** | **Health** | 124.23 ± 9.34 | 107.16 ± 35.31 (13.73%) | 0.2617 | 0.3016 (ns) | 81.38 ± 7.50 (34.49%) | 0.0863 | 0.1467 (ns) |
|  |  | **Gingivitis** | 95.29 ± 31.58 | 61.64 ± 2.35 (35.31%) | 0.1753 | 0.2338 (ns) | 71.11 ± 1.85 (25.38%) | 0.2278 | 0.2816 (ns) |
|  |  | **Periodontitis** | 98.96 ± 16.02 | 62.16 ± 1.14 (37.19%) | 0.1014 | 0.1567 (ns) | 82.19 ± 2.14 (16.95%) | 0.1686 | 0.2271 (ns) |
|  | **LPS Controls** | **E. coli LPS** | 28.88 ± 4.65 | 23.30 ± 4.24 (19.34%) | 0.2687 | 0.3071 (ns) | 7.14 ± 1.64 (75.28%) | 0.0311 | 0.0932 (ns) |
|  |  | **P. gingivalis LPS** | 13.42 ± 3.11 | 7.11 ± 10.05 (47.06%) | 0.2102 | 0.2623 (ns) | 0.00 ± 0.00 (100.00%) | 0.0517 | 0.1256 (ns) |
| IL-6 | **Subgingival samples** | **Health** | 3.76 ± 1.75 | 0.00 ± 0.00 (100.00%) | 0.1014 | 0.1567 (ns) | 0.00 ± 0.00 (100.00%) | 0.1014 | 0.1567 (ns) |
|  |  | **Gingivitis** | 7.21 ± 0.00 | 3.76 ± 1.75 (47.85%) | 0.1098 | 0.1606 (ns) | 1.26 ± 1.78 (82.52%) | 0.0664 | 0.1309 (ns) |
|  |  | **Periodontitis** | 9.38 ± 0.18 | 3.76 ± 1.75 (59.89%) | 0.0624 | 0.1309 (ns) | 2.52 ± 0.00 (73.12%) | 0.0058 | 0.0499 * |
|  | **Saliva samples** | **Health** | 7.87 ± 0.60 | 5.00 ± 0.00 (36.43%) | 0.0469 | 0.1226 (ns) | 3.76 ± 1.75 (52.19%) | 0.0624 | 0.1309 (ns) |
|  |  | **Gingivitis** | 8.92 ± 0.47 | 4.41 ± 0.84 (50.62%) | 0.0643 | 0.1309 (ns) | 7.37 ± 0.22 (17.43%) | 0.0357 | 0.1011 (ns) |
|  |  | **Periodontitis** | 10.22 ± 1.37 | 3.76 ± 1.75 (63.21%) | 0.1049 | 0.1579 (ns) | 5.00 ± 0.00 (51.08%) | 0.0585 | 0.1309 (ns) |
|  | **LPS Controls** | **E. coli LPS** | 6.11 ± 1.56 | 1.91 ± 2.69 (68.80%) | 0.0599 | 0.1309 (ns) | 1.26 ± 1.78 (79.36%) | 0.1445 | 0.2027 (ns) |
|  |  | **P. gingivalis LPS** | 0.00 ± 0.00 | 0.00 ± 0.00 | ns | ns | 0.00 ± 0.00 | ns | ns |
| IL-10 | **Subgingival samples** | **Health** | 1.69 ± 0.00 | 1.69 ± 0.00 (0.00%) | ns | ns | 0.00 ± 0.00 (100.00%) | ns | ns |
|  |  | **Gingivitis** | 2.21 ± 0.74 | 0.84 ± 1.20 (61.85%) | 0.0730 | 0.1354 (ns) | 0.84 ± 1.20 (61.85%) | 0.0730 | 0.1354 (ns) |
|  |  | **Periodontitis** | 3.20 ± 0.64 | 1.69 ± 0.00 (47.10%) | 0.0935 | 0.1523 (ns) | 1.71 ± 0.04 (46.32%) | 0.0900 | 0.1511 (ns) |
|  | **Saliva samples** | **Health** | 2.67 ± 1.39 | 1.69 ± 0.00 (36.70%) | 0.2500 | 0.2906 (ns) | 1.37 ± 1.94 (48.69%) | 0.0928 | 0.1523 (ns) |
|  |  | **Gingivitis** | 1.69 ± 0.00 | 0.84 ± 1.20 (50.00%) | 0.2500 | 0.2906 (ns) | 1.69 ± 0.00 (0.00%) | ns | ns |
|  |  | **Periodontitis** | 2.21 ± 0.74 | 1.69 ± 0.00 (23.70%) | 0.2500 | 0.2906 (ns) | 1.69 ± 0.00 (23.70%) | 0.2500 | 0.2906 (ns) |
|  | **LPS Controls** | **E. coli LPS** | 0.00 ± 0.00 | 0.00 ± 0.00 | ns | ns | 0.00 ± 0.00 | ns | ns |
|  |  | **P. gingivalis LPS** | 0.00 ± 0.00 | 0.00 ± 0.00 | ns | ns | 0.00 ± 0.00 | ns | ns |
| TGF-beta | **Subgingival samples** | **Health** | 937.60 ± 14.28 | 859.95 ± 62.15 (8.28%) | 0.1936 | 0.2526 (ns) | 880.20 ± 43.70 (6.12%) | 0.1974 | 0.2526 (ns) |
|  |  | **Gingivitis** | 917.95 ± 14.78 | 919.00 ± 34.08 (-0.11%) | 0.5097 | 0.5501 (ns) | 884.55 ± 3.18 (3.64%) | 0.0766 | 0.1354 (ns) |
|  |  | **Periodontitis** | 948.30 ± 35.78 | 938.70 ± 45.40 (1.01%) | 0.1962 | 0.2526 (ns) | 939.45 ± 12.94 (0.93%) | 0.4200 | 0.4606 (ns) |
|  | **Saliva samples** | **Health** | 781.45 ± 63.71 | 848.45 ± 3.75 (-8.57%) | 0.8204 | 0.8517 (ns) | 842.40 ± 33.66 (-7.80%) | 0.8932 | 0.9065 (ns) |
|  |  | **Gingivitis** | 878.60 ± 61.38 | 882.80 ± 26.02 (-0.48%) | 0.5216 | 0.5586 (ns) | 958.30 ± 7.21 (-9.07%) | 0.8574 | 0.8767 (ns) |
|  |  | **Periodontitis** | 934.40 ± 66.04 | 921.15 ± 3.89 (1.42%) | 0.4167 | 0.4606 (ns) | 934.90 ± 15.56 (-0.05%) | 0.5045 | 0.5489 (ns) |
|  | **LPS Controls** | **E. coli LPS** | 891.05 ± 46.46 | 863.95 ± 1.91 (3.04%) | 0.2739 | 0.3104 (ns) | 876.80 ± 57.56 (1.60%) | 0.1603 | 0.2202 (ns) |
|  |  | **P. gingivalis LPS** | 957.45 ± 15.06 | 835.05 ± 45.33 (12.78%) | 0.1068 | 0.1579 (ns) | 909.85 ± 27.51 (4.97%) | 0.1795 | 0.2370 (ns) |
