# Supplementary material for: Modulation of LPS-associated virulence activity for reduction of periodontal inflammatory burden
Source: Front Microbiol. 2026 Jan 30;17:1728315. doi: 10.3389/fmicb.2026.1728315 (PMC12902939; doi:10.3389/fmicb.2026.1728315)
Supplement: Supplementary file 2 [file Image_1.pdf]

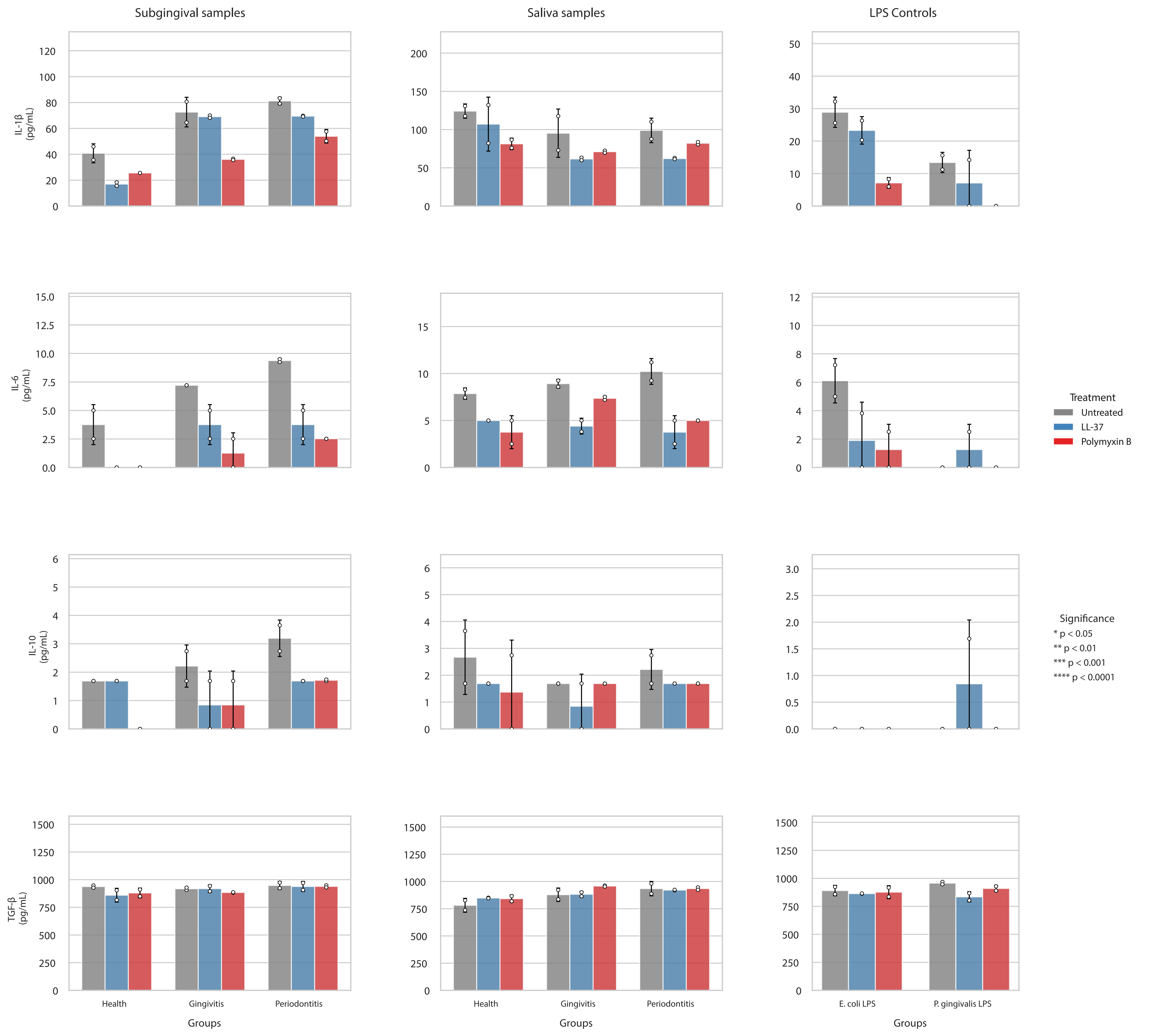

Subgingival samples
Saliva samples
LPS Controls
IL-1β (pg/mL)
IL-6 (pg/mL)
IL-10 (pg/mL)
TGF-β (pg/mL)
Health
Gingivitis
Periodontitis
E. coli LPS
P. gingivalis LPS
Groups
Treatment
Untreated
LL-37
Polymyxin B
Significance
* p < 0.05
** p < 0.01
*** p < 0.001
**** p < 0.0001
